# Canada1Water digital terrain model with bathymetry dataset

**DOI:** 10.1016/j.dib.2026.112878

**Published:** 2026-05-22

**Authors:** Eric D. Kessel, Steve K. Frey, Hazen A.J. Russell

**Affiliations:** a Aquanty Inc., 600 Weber St. N., Unit B, Waterloo, ON N2V 1K4, Canada; b Geological Survey of Canada, 601 Booth St. Ottawa, ON K1A 0E8, Canada

**Keywords:** Digital elevation model, Numerical modelling, Hydrogeology, Lake depth

## Abstract

National integrated water modelling of Canada required a (DEM) Digital Elevation Model for all continental Canada at a single resolution, with bathymetry, and inclusion of transboundary watersheds with the United States. Such a model was not available and hence the Canada1Water project developed a Digital Terrain Model (DTM) that integrates high resolution elevation data from derivative Arctic (2 m) and NASADEM (30 M) DEM with open-source bathymetry data for lakes ≥10 km^2^. The DTM was brought to a standard datum and projection, resampled to a standard horizontal scale, and corrected for void issues and canopy biases. The result is the 1-arcsecond grid (∼20 m) Canada1Water Digital Terrain Model (C1W-DTM). The new DTM provides a standard elevation datum for all subsurface modelling as well as a constraint for the surface hydraulic flow system. A seamless DTM with lake Bathymetry was essential to support the control volume HydroGeoSphere modelling environment being used. This dataset supports a wide range of applications requiring a DTM with lake bathymetry thus enabling more accurate and informed decision-making.

Specifications Table

**Table:** 

Subject	Earth & Environmental Sciences
Specific subject area	Gridded digital terrain model to support national scale integrated surface-water groundwater hydrological numerical modelling.
Type of data	Stacked Geotiff gridded rasters at ∼ 20m xy resolution available in 10×10-degree macro-tiles. Band 1 is the digital surface model. Band 2 is the digital elevation model. Band 3 is the digital terrain model with lake bathymetry.
Data collection	Several publicly available digital elevation models (DEM) datasets across Canada have been harmonized to produce a standardized, hydrogeologically robust terrain surface. South of 60°N latitude, the base elevation dataset is derived from the NASA’s NASADEM product at 1-arcsecond resolution (30m at the equator but ∼20m in Canada), while north of 60°N, elevations are sourced from the High-Resolution Digital Elevation Model (HRDEM) at 2 m resolution, down-sampled to match the NASADEM resolution. All DEMs were reprojected to a common horizontal projection and referenced to the CGVD2013a vertical datum. To address known limitations in the source DEMs, several corrections were applied. A vegetation canopy height model was developed for regions south of 60°N and subtracted from the NASADEM to approximate bare-earth elevations; vegetation effects were assumed negligible and therefore not corrected in northern regions above 60°N. Voids and artefacts in the HRDEM above 60°N were filled using the Copernicus GLO-30m digital surface model (DSM). To further enhance applicability for hydrogeological modelling, bathymetric data for lakes larger than 10 km² were compiled from publicly available sources. Real bathymetry was available for several of the largest larges which were integrated directly. Estimated lake bathymetry was generated for Where direct measurements were unavailable, estimated bathymetry was generated and integrated. These bathymetric surfaces were “burned” into the elevation model to ensure realistic representation of below-water terrain. The final dataset is the Canada1Water Digital Terrain Model (C1W-DTM), a seamless, void-free, and hydrologically conditioned 1-arcsecond (∼20 m) consisting of 3 individual bands representing the DSM, DEM and DTM, respectively. The C1W-DTM was designed to support national-scale water resource applications.
Data source location	This dataset is publicly available through the Canada1Water (C1W) community on Zenodo (https://zenodo.org/communities/c1w/). It is also available through the Canada1Water data portal which (https://www.canada1water.ca/) provides a national hydrological modelling framework to support integrated groundwater–surface water simulations across Canada. The Canada1Water hydrological domain spans approximately 12 × 10^6^ km² and encompasses the main continuous continental Canadian landmass, including Baffin Island, Island of Newfoundland, and Vancouver Island, as well as transboundary watersheds with the United States. The portal hosts standardized, harmonized datasets and model domains intended for consistent national-scale hydrological analyses and numerical modelling applications.
Data accessibility	Repository name: https://portal.canada1water.ca/ https://zenodo.org/communities/c1w/ Data identification number: https://doi.org/10.5281/zenodo.17807459 Direct URL to data: https://zenodo.org/records/17807459 https://portal.canada1water.ca/#/c1w-downloads Instructions for accessing these data: The latitude and longitude can be entered to retrieve a zipped file of the 10×10-degree Geotiff tiled dataset. Tilename structure: n{latitude}w{longitude} Filename structure: c1w_DEMv2_{tilename}_10deg_macrotile.tif
Related research article	None.

## 1 Value of the Data

• The C1W-DTM provides seamless, national-scale coverage across Canada, including transboundary watersheds that extend into the United States. It is distributed at a spatial resolution of 1 arcsecond, which corresponds to approximately 30 m at the equator but increases in spatial detail to ∼ 20 m across most of Canada due to latitudinal convergence of meridians.
• All elevation values in the C1W-DTM are referenced to the Canadian Geodetic Vertical Datum of 2013 (CGVD2013a), ensuring consistency with modern geodetic standards used across Canada. This vertical datum alignment allows the dataset to be reliably integrated with other national-scale geospatial and hydrometric datasets, reducing vertical inconsistencies that can otherwise introduce errors in modeling workflows.
• The dataset includes three primary elevation bands representing different surface conditions. These consist of a Digital Surface Model (DSM), which captures elevations including vegetation and built features; a Digital Elevation Model (DEM), representing a partially filtered surface; and a Digital Terrain Model (DTM), which reflects a hydrologically conditioned bare-earth surface with integrated bathymetry. This multi-band structure allows users to select the most appropriate surface representation depending on their application.
• In addition to the primary elevation layers, the dataset includes derivative bands that provide further analytical value. A lake bathymetry layer is included, representing both observed and estimated depth information for large waterbodies, while a canopy height model is provided to quantify vegetation height and support the derivation of bare-earth elevations in forested regions. These derivative products enhance the dataset’s usability for ecological, hydrological, and land surface modeling applications.
• To facilitate efficient storage, distribution, and processing, the dataset is partitioned into 57 macro-tiles, each covering a 10×10-degree geographic extent. This chunking approach allows users to download and work with manageable spatial subsets while maintaining consistency across tile boundaries, making it well suited for both regional and continental-scale analyses.

## 2 Background

Canada1Water (C1W) is an initiative focused on modelling Canada’s complete water cycle, with particular attention to groundwater–surface water interactions. This requires a national scale, bare-earth DEM that includes bathymetry (hereafter referred to as the C1W Digital Terrain Model or C1W-DTM). The C1W model domain includes mainland Canada, U.S. transboundary watersheds, and Baffin Island. To support continental scale hydrological–hydrogeological modelling of this domain, a seamless topographic boundary is essential. However, no single DEM source met all the required characteristics for geographic extent, type, scale, registration, canopy-correction and representation of hydraulic features. Furthermore, a DEM with 1-arc second horizontal scale was required to support modelling and it was necessary to have accurate registration with Earth Observation datasets.

**Fig. 1:**
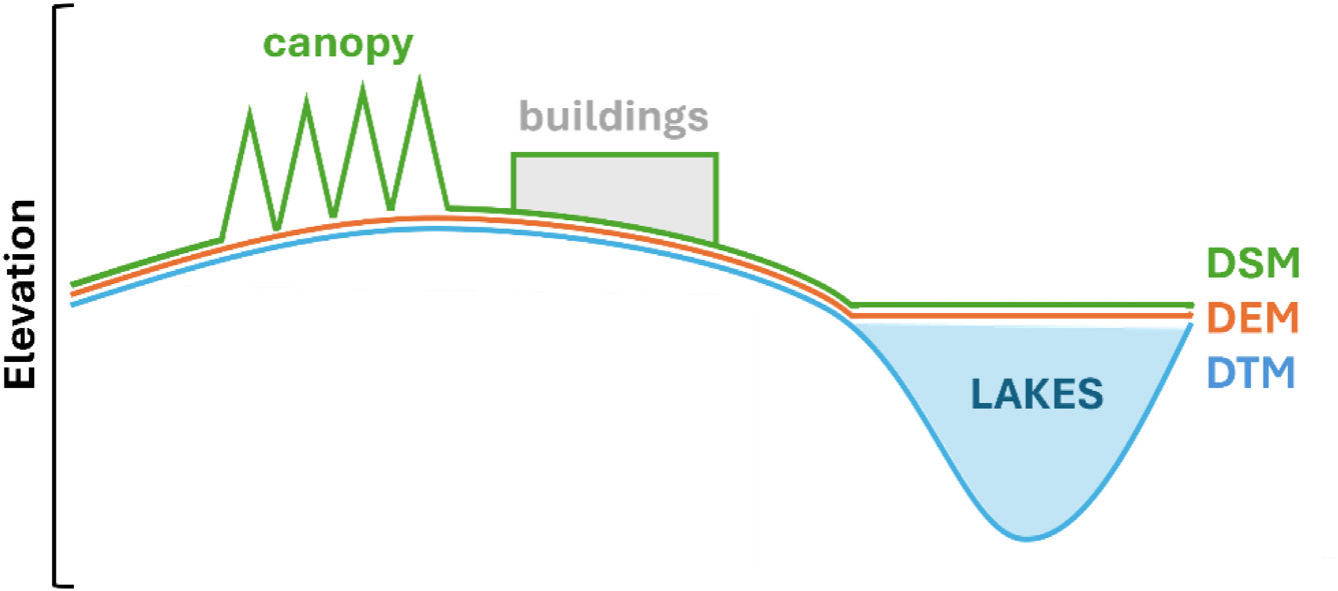
Conceptual diagram of digital surface (DSM), elevation (DEM) and Terrain Models (DTM). Note surface includes vegetation and human infrastructure (buildings, roads, etc.).

The proliferation of DEMs in the past 20 years has resulted in an abundance of products with various parameters and terminologies, often causing confusion. In this paper, we adopt the conventions of Guth et al. (2021) [1], whereby the term DEM is used as a generic term for any digital representation of ground surface. A Digital Surface Model (DSM) is a DEM that represents the surface elevation, including vegetation, anthropogenic structures (e.g., buildings, roads, powerlines), and water surfaces. In contrast, a Digital Terrain Model (DTM) is a DEM that represents the bare land surface and incorporates lake and/or ocean bathymetry (Fig. 1).

The Canadian landscape is represented by a variety of publicly available DEM models generated using diverse methodologies, spanning a range of scales and processed through a range of workflows. Most datasets are available at regional or provincial scales, with only two or three offering near-national coverage [2,3]. The purpose and scale of DEM creation influence the choice of collection methods (e.g., LiDAR, radar, optical, aerial, satellite), horizontal and vertical resolution, accuracy, and post-processing workflows (e.g., Guth et al., 2021).

National Canadian DEM coverage is limited to the Canadian Digital Elevation Dataset (CDED), Canadian Digital Elevation Model (CDEM) [2], and the Medium Resolution DEM (MDREM) [3]. The CDED and CDEM datasets were both derived from contour lines generated through photogrammetric surveys conducted in the 20th Century. Their horizontal and vertical resolution limitations, coupled with the lack of registration with satellite-derived products, have rendered these datasets outdated. Canada’s High Resolution DEM (HRDEM) [4] built from 1 m resolution LiDAR projects is best in class, but current coverage is only ∼15% of the Canadian landscape.

A successor product, the Canadian Digital Surface Model (CDSM) was derived from the 1-arcsecond (30 m) Shuttle Radar Topography Mission [5]. However, its coverage is restricted to areas south of 60°N. Since the initiation of the C1W-DTM project, Natural Resources Canada has released the 30 m Medium Resolution DEM (MRDEM) [2]. The CDED and CDEM are no longer fit for purpose, and CDSM has been superseded by the NASADEM. Unfortunately, the MRDEM was not available when work started on the C1W DTM.

For hydrological process studies, DEMs are often reprocessed to make them hydrologically conditioned. While there is no single, agreed upon definition for hydrologically conditioned DEMs, the processing typically involves two main steps i) ensure continuity of water flow over the DEM (land) surface, often through pit removal and ii) hydrological flow enforcement.

For C1W, hydrological conditioning of the DTM was not necessary, as topographic elevation adjustments were made directly in the HydroGeoSphere (HGS; Aquanty Inc., 2015) model’s finite element mesh using HGS’s downgradient flow enforcement functionality. This approach allows for greater flexibility when integrating updated DEMs, high-resolution regional scale DEMs, and updated river vector data into the C1W modelling framework since the HGS model is not sensitive to hydrological conditioning procedures and the DEM is independent of river geometry. HGS does require lake bathymetry to be stitched into the DEM where groundwater– lake interactions are of interest in the analysis, as is the case for C1W. HGS is a mass conservative model wherein the volume of water in the lakes is a key component of the analysis, and because HGS tracks water exchange between the groundwater system and the surface water/lake system based on 3-dimensional pressure gradients.

The objective of this activity was to create a harmonized DTM for the C1W model domain to support HGS modelling and to make the product publicly available. This document outlines the process of compiling and reprocessing DEMs from several data sources covering the Canadian landscape. It details the methodologies, data sources, processing techniques, and quality assurance measures employed to create the C1W-DTM. The final product is a void-free, national scale DTM with lake bathymetry for lakes larger than 10 km^2^.

## 3 Data Description

**Fig. 2:**
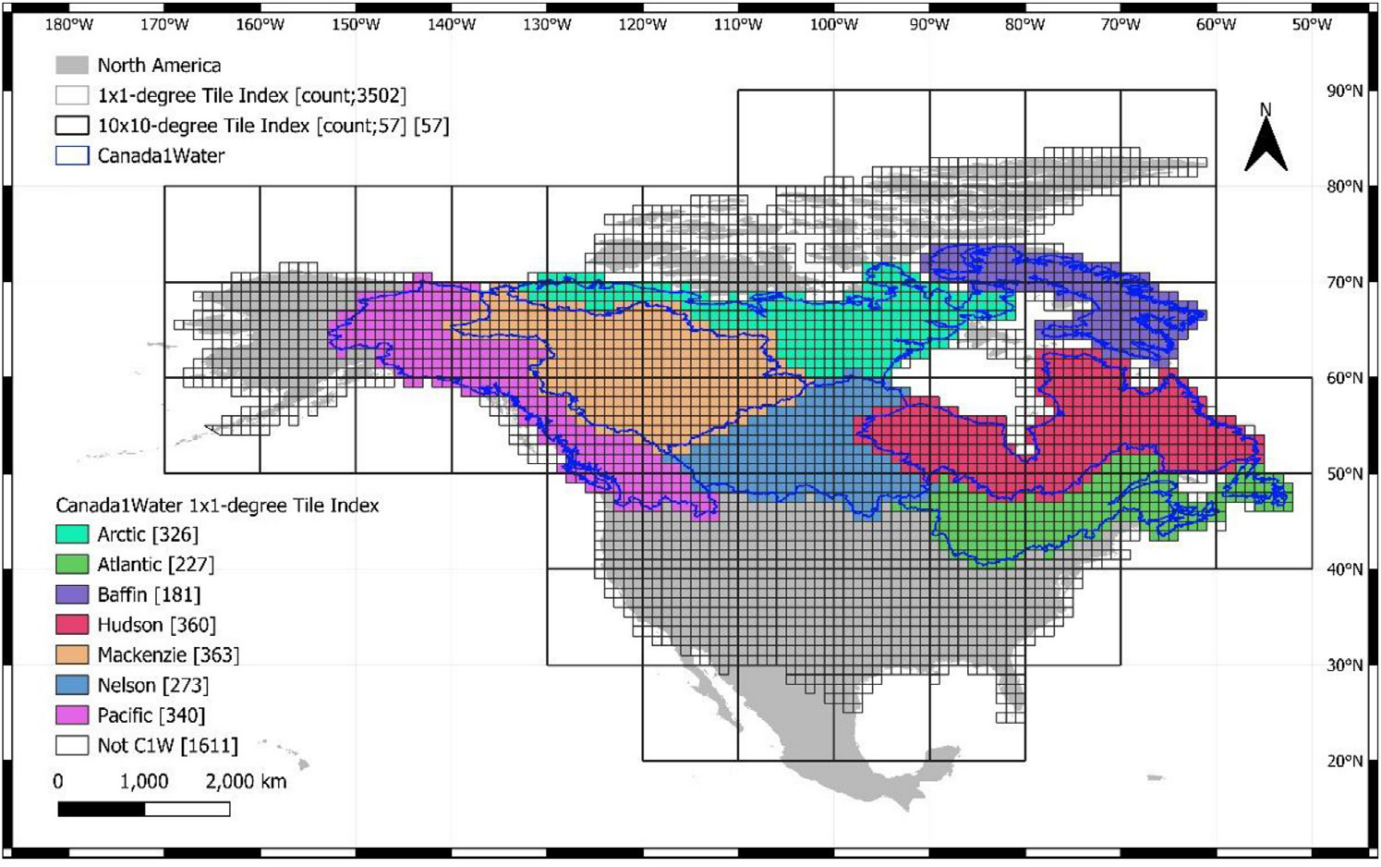
Dataset coverage over the Canada1Water model domain with the 57 macro-tiles extents illustrated.

All dataset tiles were joined into a 1-arcsecond horizontal resolution mosaic with three bands of data: 1) DSM with voids present, 2) DEM after canopy correction and voids filled and 3) DTM after lake bathymetry and voids filled (Fig. 7). The final mosaic was divided (chunked) into 57 individual 10×10-degree macro-tiles for ease of distribution (Fig. 2). The canopy model and lake bathymetry are implicitly present in the 3 bands of data. The canopy can be extracted by subtracting band 2 from band 1, and the bathymetry can be extracted by subtracting band 2 from band 3. The mosaic's horizontal projection is EPSG-4326, with the vertical datum CGVD2013a, and the data format is integer-only (INT16).

## 4 Experimental Design, Materials and Methods

### 4.1 Data coverages for Canada

#### 4.1.1 Digital surface and elevation models

Seven DEMs, available from Canadian government, US government and commercial sources, were used to support development of the C1W-DTM (Table 1). The High-Resolution Digital Elevation Model (HRDEM) [3] and MRDEM [2] are two national Canadian government datasets. The NASADEM [5] and MERIT [6] both include SRTM data as the core dataset with subsequent reprocessing. Copernicus provides a global DSM [7], with the FABDEM [8] being a derivative product, which is regarded to be best in class.

**Table 1:** List of DEM datasets available for C1W-DTM mosaic.

Name	Type	Resol-ution	Coverage	Vertical Datum	Vertical Accuracy	Sensor Type	Reference	Source Date	Data
HRDEM	DTM	1m	variable Canada	CGVD2013	0-1m	LiDAR	NRCan, 2022a	ongoing	float
HRDEM /Arctic DEM	DSM	2m	Arctic, above N60° latitude	CGVD2013 WGS84	0-2m	optical stereo imagery	NRCan, 2022a; Porter et al., 2023	2007 and 2021	float
MRDEM	DEM	30m	Canada	CVGD2013	0-10m	Radar	NRCan, 2024	2011-2015	float
NASADEM	DSM	30m	Global, below N60° latitude	EGM96	0-16m	Radar	NASA, 1997	2000	integer
MERIT	DTM	90m	Global	EGM96	0-16m	Radar	Yamazaki et al., 2017	2000	integer
Copernicus Cop30m	DSM	30m	Global	EGM2008	0-10m	Radar	AIRBUS, 2020	2011-2015	float
FABDEM	DEM	30m	Global	EGM2008	0-10m	Radar	Hawker et al., 2022	2011-2015	float

##### 4.1.1.1 ArcticDEM USA

ArcticDEM [9] is a 2 m DSM derived from optical stereo imagery for areas above 60°N latitude and is the source data for the HRDEM 2 m DSM (see below). ArcticDEM is in the WGS84 ellipsoid vertical projection (non geoid).

##### 4.1.1.2 Copernicus COP-DEM-GLO-30-DTED

Copernicus [7] is a 30 m DSM derived from the 12 m resolution German TanDEM-X mission collected between 2010 and 2015. Datum is WGS84-G1150 (EPSG 4326) and vertical datum is EGM2008 (EPSG 3855). Copernicus is considered the current gold standard in 30 m global datasets, outperforming SRTM, NASADEM, ASTER GDEM and ALOS AW3d30.

##### 4.1.1.3 FABDEM forest and building removed DEM

Base DSM model is Copernicus Global 30 m with post processing using machine learning to remove canopy and building biases to create the FABDEM (Forest and building removed digital elevation model) [8]. Availability is restricted by commercial license for reproduction.

##### 4.1.1.4 HRDEM 2 m Canada

DSM available above the N60 latitude for the Canadian Arctic, the HRDEM [4] was based on the ArcticDEM [9] with additional post-processing by the Canada Centre for Mapping and Earth Observation (CCMEO) to create a 2-meter Digital Surface Model (DSM) covering a geographic region of approximately 4.6 million km^2^. HRDEM uses the North American 1983 CSRS (NAD 83 CSRS) datum, and the vertical datum is CGVD2013a.

##### 4.1.1.5 HRDEM 1 m Canada

A mosaic of LiDAR projects, municipal, provincial, etc., resolved at 1 m resolution for select project areas across Canada, mostly below 60° N latitude. HRDEM [4] uses the North American 1983 CSRS (NAD 83 CSRS) datum, and the vertical datum is CGVD2013a.

##### 4.1.1.6 MERIT: multi-error-removed improved-terrain DEM

The MERIT [6] a downscaled derivative of SRTM (90 m xy resolution) with noise-, elevation bias- and canopy- corrections. SRTM v2.1 South of 60° latitude, ALOS AW3D North of 60° latitude, Vertical datum EGM96.

##### 4.1.1.7 MRDEM

MRDEM-30-DSM [3] is based on the GLO-30 DSM. The modifications to create the MRDEM-30- DSM include reprojection to NAD83(CSRS) Canada Atlas Lambert (EPSG:3979), resampling to a 30 meters resolution grid, and vertical datum transformation from EGM2008 to CGVD2013.

##### 4.1.1.8 NASADEM *global digital elevation model*

NASADEM [5] is the successor of SRTM 1 Arc-Second Global which is available between the 60° N and 56° S latitude, notable improvements include reprojection to the vertical datum EGM96, absolute elevation bias correction, interpolation, and void filling.

Canadian HRDEM products [4] are developed from light detection and ranging (LiDAR) data and the ArcticDEM [9]. The LiDAR method measures the DSM (top-of-canopy) and DTM (through-canopy) allowing for canopy bias corrections resulting in a very high vertical precision in absolute elevation. Due to the vast size of Canada (∼10 million km^2^), only about ∼15% of the country has completed 1 m LiDAR coverage. For instance, complete coverage is available for New Brunswick and Alberta, whereas Ontario and Quebec only have coverage for southern areas, largely the St Lawrence Lowland region. Generally, LiDAR data is available in urban, agricultural, and flood prone areas.

The HRDEM [4] data north of 60°N is derived from the ArcticDEM project [9], and is a DSM derived from optical satellite stereo imagery. With a similar 2 m horizontal resolution to LiDAR, the ArcticDEM DSM however has a lower vertical accuracy and precision, and it includes vegetation along with snow drifts. Currently, there are no effective methods to address these biases north of 60°N. These biases are generally considered minor, as much of the area north of 60°N is beyond the tree line. Voids exist within the HRDEM, requiring void filling [10].

NASADEM [4] is an improved iteration of the SRTM at 1-arcsecond horizontal resolution and is void free. The Multi-Error-Removed Improved-Terrain DEM (MERIT) [6] addresses canopy, urban, and noise biases inherent in the SRTM, making it an important improvement, although it is down-sampled to a 90 m horizontal resolution. Copernicus DSM (COP30m) [7] is also a void-free 30 m horizontal resolution DSM with global coverage. It is a more recent 2015 project with improved technical instrumentation over the SRTM, resulting in a slightly improved resolution DSM. Advances in machine learning to predict and correct for canopy and urban building biases have been applied to the COP30m in the new Forests and Building Removed (FABDEM) [8], revealing a true bare-earth DEM; however, it does not include lake bathymetry.

### 4.2 Lake bathymetry

Digital lake bathymetry was incorporated into the C1W-DTM only for lakes larger than 10 km², with the level of detail dependent on data availability. High-resolution, observation-based bathymetry is available for a small number of very large Canadian lakes, including the Great Lakes [11], Lake Winnipeg [12]), Great Bear Lake [13], and Great Slave Lake [14]. While additional bathymetric information exists from provincial sources for smaller lakes (e.g., navigational or recreational lakes), these data are typically not available in a consistent digital format and were therefore not included.

**Fig. 3:**
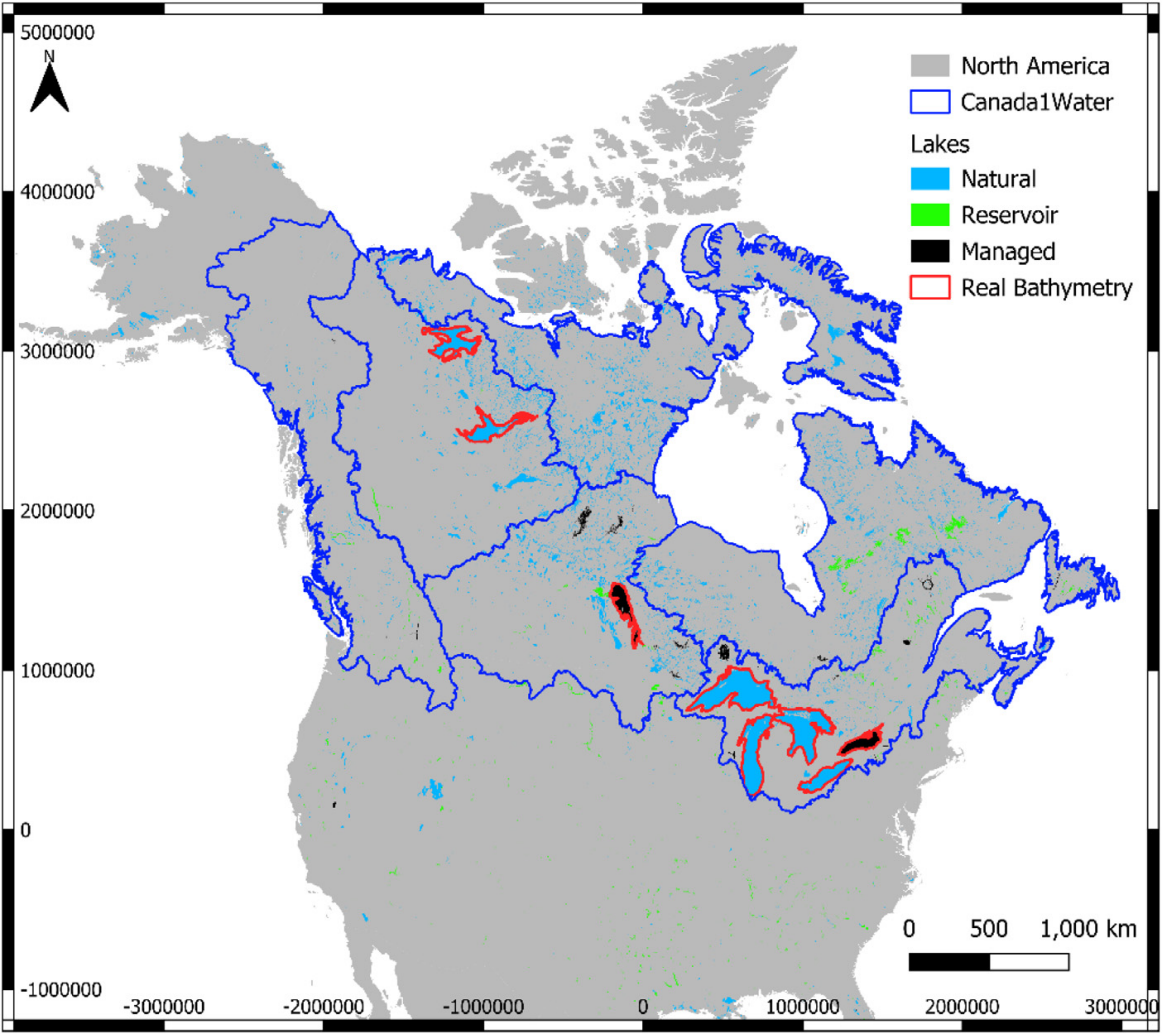
Map of lakes ≥10 km^2^ for which digital bathymetric files were available from government sources (red outline) or Khazaei et al., (2022). Dark blue outlines are the seven C1W water basin model domains.

For all remaining lakes ≥10 km² lacking detailed surveys, bathymetry was derived from the Global Lake Bathymetry (GLOBathy) dataset [15] (Fig. 3). GLOBathy predicts maximum lake depth using shoreline length, surface area, reported volume, surface elevation, and watershed characteristics as predictor variables [16]. In total, eight large lakes were represented using detailed bathymetric datasets, and an additional 7,020 lakes ≥10 km² were represented using GLOBathy-derived bathymetry.

### 4.3 Methodology

**Fig. 4:**
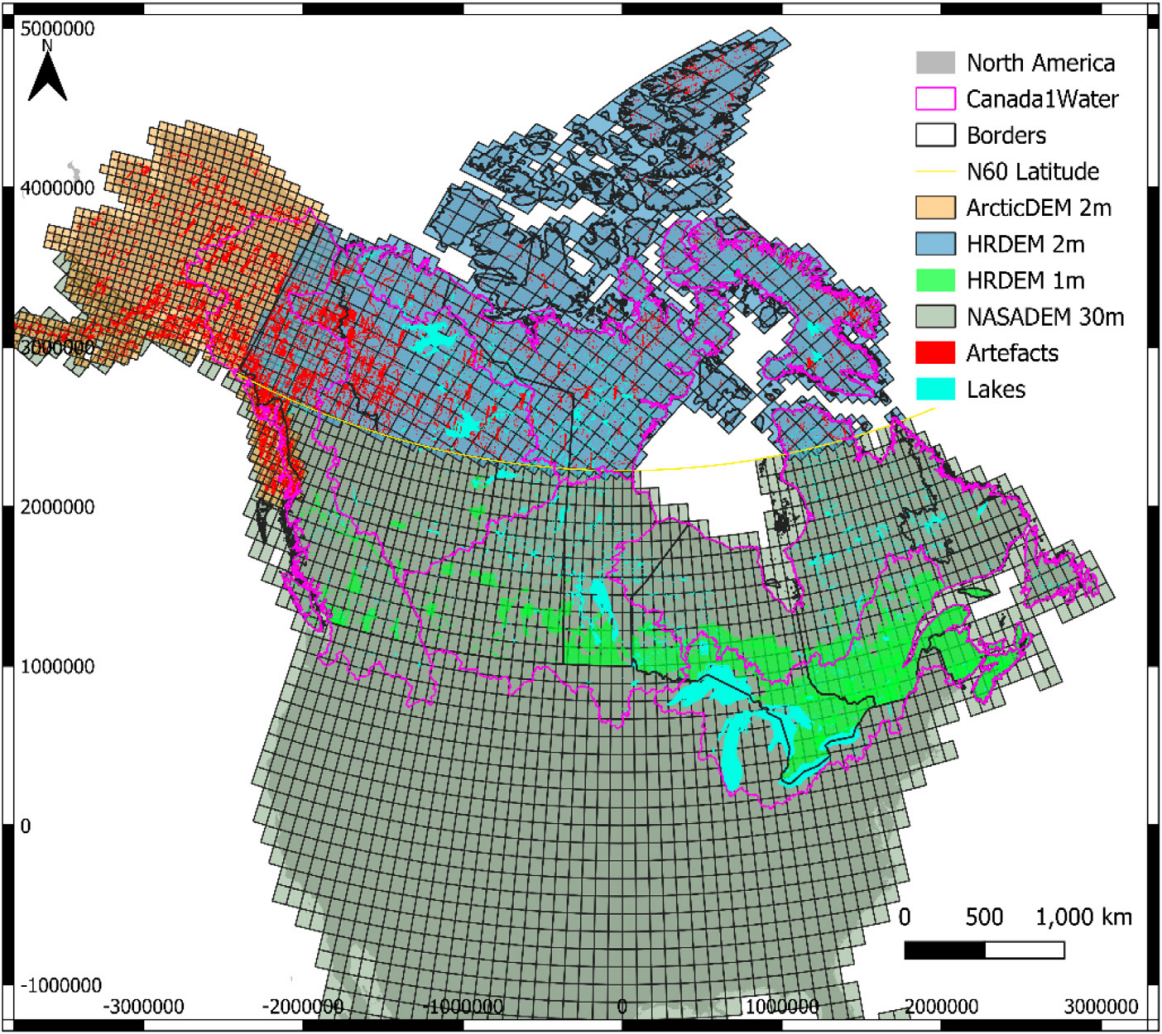
Footprints of the different datasets used in the C1W-DTM. LiDAR (1 m horizontal resolution as of June 2024) used for validation are in green.

**Fig. 5:**
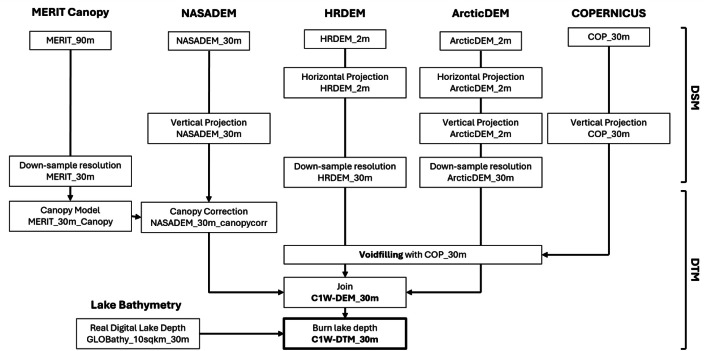
Data workflow for the DSM-to-C1W-DTM for all datasets included.

Constructing a suitable DTM for the C1W model domain required integration of multiple DSM products with canopy bias corrections, inclusion of lake bathymetry and use of additional DEM or DSM products to infill artifacts such as voids. Three primary datasets were used: NASADEM for areas south of 60° N latitude, the arctic HRDEM for areas north of 60° N latitude, and the ArcticDEM for Alaska (Fig. 4). As HRDEM coverage ends at the Canada-USA border, the ArcticDEM was needed to complete the transboundary watersheds into Alaska. Voids and artefacts within the HRDEM and ArcticDEM were filled with the COP30m DSM, whereas the NASADEM is already void-free south of 60° N latitude. All datasets were brought to a common horizontal projection, vertical datum and horizontal resolution. Voids were filled, a canopy correction was applied, and finally, lake bathymetry was subtracted to produce the DTM. The data workflow from DSM-to-DTM is detailed below and illustrated in Fig. 5.

For data processing efficiency, all datasets were divided (chunked) into either 50×50 km EPSG3414 tiles (2,500 km²) for HRDEM and ArcticDEM above 60° N latitude, or 1×1-degree WGS4326 tiles (∼12,000 km²) below 60° N latitude.

### 4.4 Horizontal projections and vertical datums

All datasets were reprojected from their native projections and datums to a common horizontal projection – the World Geodetic System (WGS4326) - and to the vertical Canadian Geodetic Vertical Datum of 2013 (CGVD2013a). Bilinear interpolation was used during reprojections, and datasets were only reprojected once to minimize errors.

### 4.5 Horizontal resampling

The higher resolution HRDEM and ArcticDEM were down-sampled from 2 m horizontal resolution to match the 1-arcsecond horizontal resolution of the NASADEM. Bilinear interpolation was used during down-sampling and was only performed once to minimize errors. For the Canadian landscape, 1-arcsecond (∼30 m at the equator) equates to approximately 20 m horizontal resolution for Canada. The lower resolution MERIT was up-sampled (bilinear) from 3-arcseconds (90 m) to match that of the 1-arcsecond NASADEM.

### 4.6 Void-filling

The COP30m was used to fill voids or artifacts, particularly in the DSM products above 60° N latitude. For more information on void and artifact issues within the 2 m HRDEM and ArcticDEM, refer to Papasodoro et al. (2023) [10]. No sophisticated image reprocessing or filtering was performed after the void filling, and edge joining offsets or seamlines do occur. It was deemed beyond the scope of this project to efficiently address seamlines. Separate raster bands exist for the DSM with and without void filling, which can be used to identify these areas if the user wishes to further process the data to reduce seamlines.

### 4.7 Canopy corrections

**Fig. 6:**
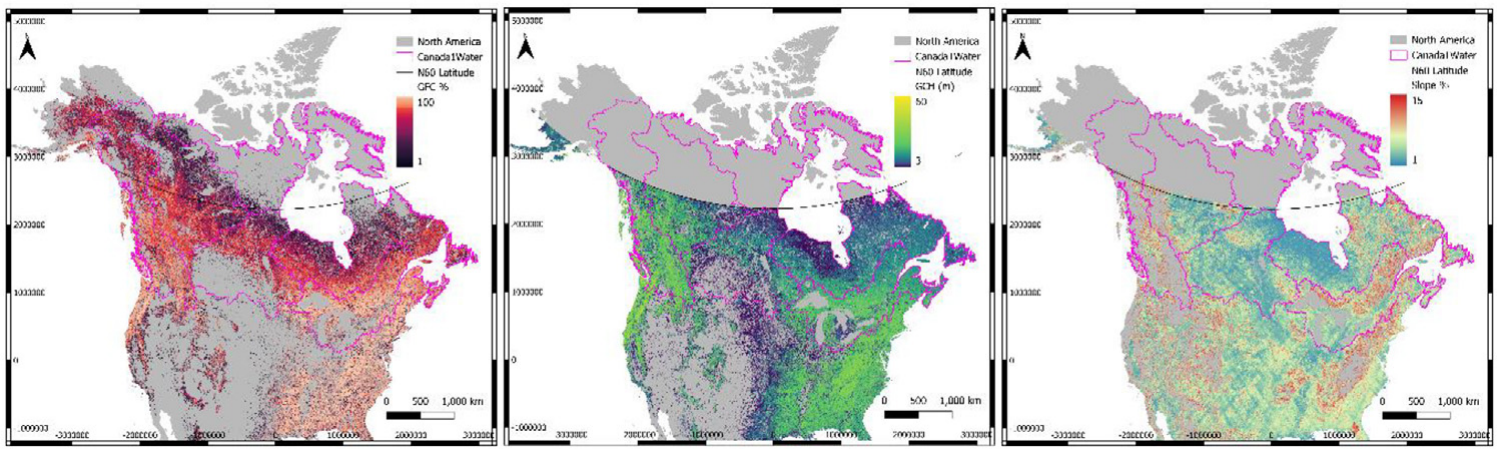
The Global Forest Cover (GFC; >0%; Hansen et al., 2013), Global Canopy Height (GCH >3 m; Lang et al., 2022), and topographic slope (<15%; NASADEM3 0m).

A pseudo canopy height model was reverse engineered by subtracting up-sampled MERIT data from the NASADEM for areas below 60° N. To help compensate for the discrepancy in resolutions between MERIT and NASADEM, a moving average window of 3×3 pixels was applied to smooth the canopy model. This helped to reduce seamlines and improve the overall performance of the canopy model (discussed later). To prevent any ‘over-correction’ or creation of undesired pits or depressions, the canopy model was confined to areas with Global Forest Coverage (GFC) [17] greater than 0%, (Global Canopy Model (GCM) [18] greater than 3 m and the topographic slope less than or equal to 15% (Fig. 6). All canopy heights outside these conditions were set to 0 m. This canopy model was validated against the highest resolution canopy heights available from LiDAR (DSM-DEM), the GCM and the FABDEM canopy model which was also back calculated by subtracting FABDEM from the COP30m.

### 4.8 Bathymetry

For the lakes >10 km^2^ in area and which the real digital lake bathymetry was not available (Fig. 3), lake depth estimation followed the approach of Khazaei et al. (2022), which assumes the deepest point occurs at the location farthest from the shoreline, based on a Euclidean distance transform. Digital lake bathymetry was generated using the *Generate_Bathymetry_Rasters.py* script [15], which utilizes the HydroLAKES [16] vector database, incorporating Canadian Vector (CanVec) and National Hydrological Dataset (NHD) waterbody polygons for Canada and the USA, respectively. After applying canopy corrections to the DSM, estimated lake depths were subtracted from the DEM to generate the DTM. Lake depths can therefore be derived by subtracting the DTM from the DEM raster bands. In total, 7,028 lakes larger than 10 km² were included in the C1W-DTM (Table 2).

**Table 2:** Count of Lakes >10 km^2^ included in C1W-DTM, divided by the seven C1W model domains.

	Number of Lakes/Reservoirs			
Model	Real Bathymetry	Estimated Bathymetry	Total Number of Lakes	Lake / Model Area
Arctic	0	1,485	1,485	6.5%
Atlantic	5	734	739	17.9%
Baffin	0	244	244	2.9%
Hudson	0	2,131	2,131	5.0%
Mackenzie	2	961	963	6.3%
Nelson	1	1,186	1,187	7.2%
Pacific	0	279	279	1.0%
Total	**8**	**7020**	**7028**	**7.0%**

### 4.9 Hydrological conditioning

Hydrological conditioning of DEMs is a critical step in hydrological modelling, but it can vary in its definition depending on the specific objectives of the modelling process. One common hydrological processing step is depression filling, which ensures that down-slope gradients are continuous. However, this step has not been completed in the C1W-DTM. Closed depressions are a natural feature of glaciated landscapes and often play a significant hydrological role, such as the Prairie potholes. Therefore, naturally occurring depressions have been preserved in the DTM, including those in the lake bathymetry.

The inclusion of lake bathymetry in the DTM allows for a more natural representation of surface water pooling and redistribution. If users of the data wish to perform depression filling or enforce downslope continuity, it is recommended that they do so on the DSM or DEM bands, rather than the DTM bands, to avoid disrupting the representation of natural depressions and lake bathymetry.

### 4.10 Data tiles and mosaic

**Fig. 7:**
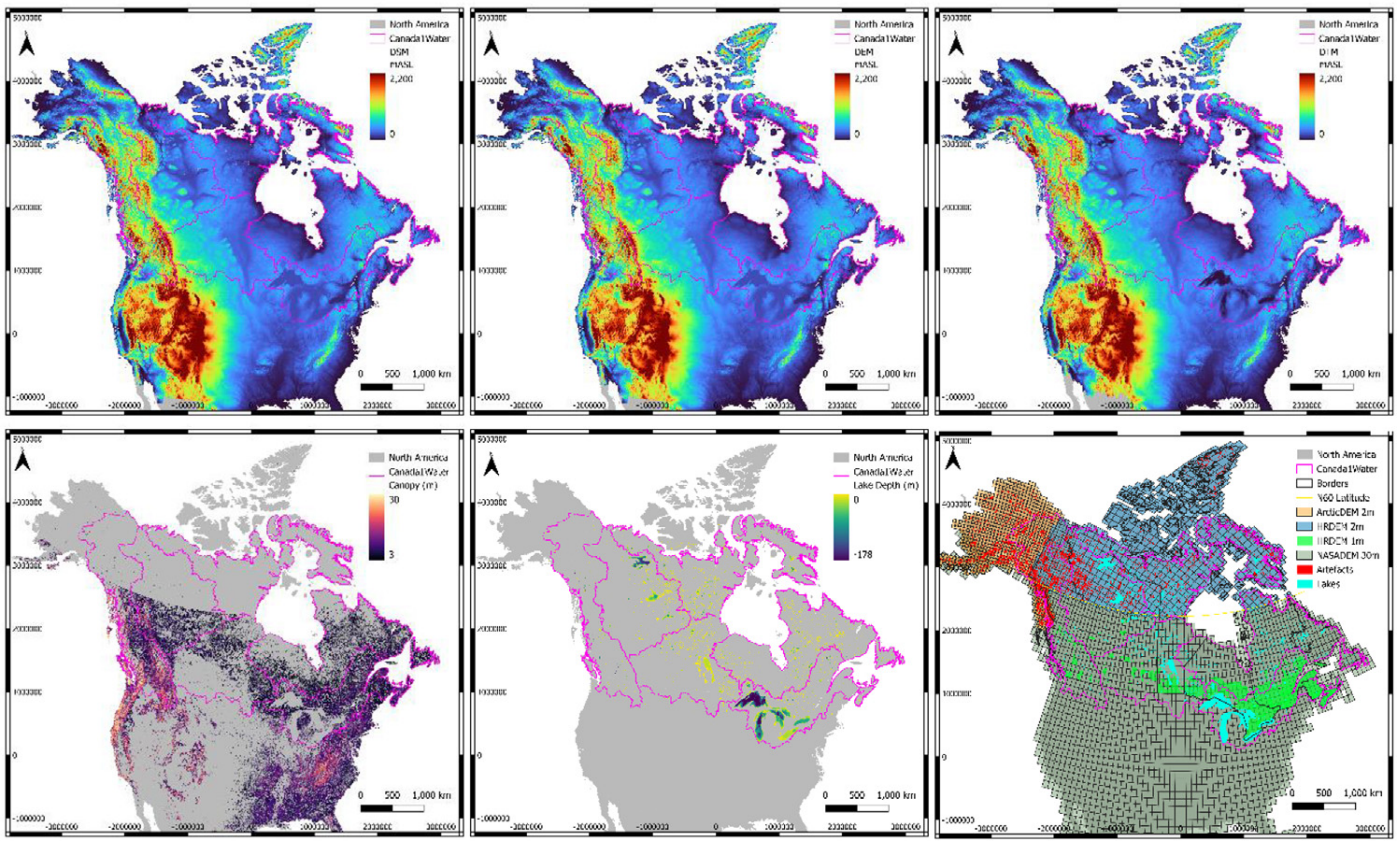
Final Canada1Water DTM Mosaic with all three raster bands for DSM, DTM and DTM with bathymetry (top left to right), along with derivative bands for the canopy model, digital lake bathymetry and dataset footprints (bottom left to right).

All dataset tiles were joined into a 1-arcsecond horizontal resolution mosaic with three bands of data: 1) DSM with voids present, 2) DTM after canopy correction and voids filled and 3) DTM after lake bathymetry and voids filled (Fig. 7). The final mosaic was divided (chunked) into 57 individual 10×10-degree macro-tiles for ease of distribution (Fig. 2). The canopy model and lake bathymetry are implicitly present in the 3 bands of data (Fig. 7). The canopy can be extracted by subtracting band 2 from band 1, and the bathymetry can be extracted by subtracting band 2 from band 3. The mosaic's horizontal projection is EPSG-4326, with the vertical datum CGVD2013a, and the data format is integer-only (INT16).

### 4.11 Evaluation

To evaluate the DEM models, the root mean squared error (RMSE) for each raster pixel in the C1W-DTM was compared to the respective benchmark LiDAR datasets, where LiDAR coverage was available (as of HRDEM release in June 2022). Most LiDAR projects are south of 60° N latitude where the C1W-DTM coverage is only based on the NASADEM and where canopy corrections were needed (Fig. 4). Thus, the term C1W-NASADEM is used to refer to this part of the C1W-DTM in this evaluation. Other DEM datasets were also included in the comparison for performance.

The RMSE measures how well the model predicts actual elevation (accuracy). However, due to the non-parametric distribution of RMSE values in DEMs compared to LiDAR benchmarks, kernel density estimation (KDE) curves were used to illustrate the RMSE distribution for each DEM dataset.

In general, KDE curves help interpret vertical precision and accuracy:

• Higher precision datasets exhibit RMSE density curves with a narrow distribution range.
• Higher accuracy datasets have mean RMSE values closer to 0.
• Lower precision datasets display wider distribution curves with higher mean RMSE values.

First, the canopy corrections applied to generate the DTM were evaluated, followed by a separate assessment of the predicted elevation of the DTM.

### 4.12 Evaluation of canopy corrections

**Fig. 8:**
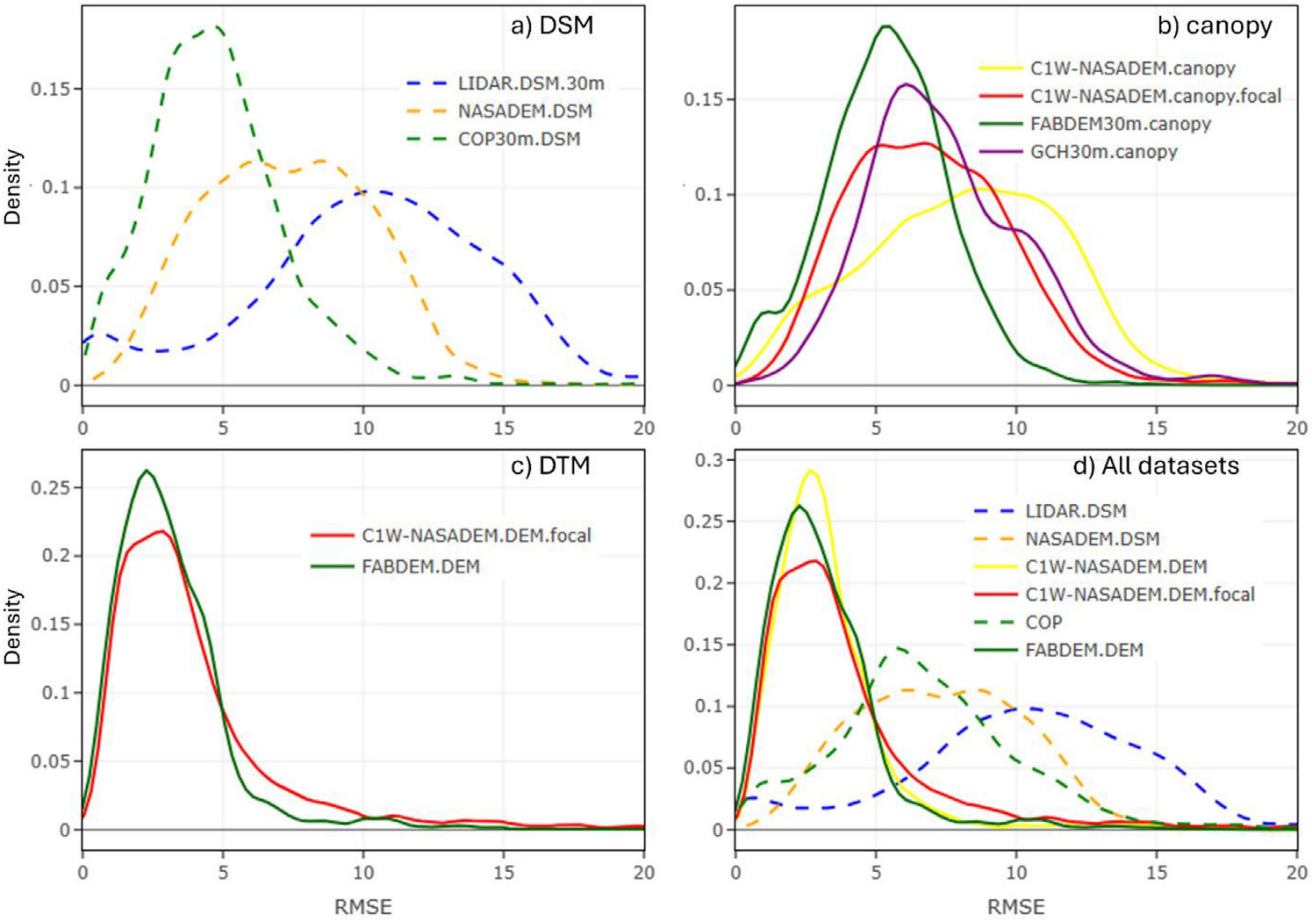
A comparison of the canopy models for forested/vegetated areas only (canopy >3 m). A) DSMs compared to the LiDAR top-of canopy. B) Canopy models compared to the LiDAR canopy heights. C) DTMs compared to the LiDAR with ground surface (through-canopy). D) All datasets are compared to the LiDAR ground surface (through-canopy). DSM are dashed lines and DTM are solid lines

To evaluate the performance of the pseudo-canopy correction applied to the C1W-DTM, the C1W-NASADEM canopy heights were compared to corresponding: i) LiDAR canopy heights (i.e., top-of-canopy minus through-canopy elevation), ii) FABDEM canopy heights (COP30m minus FABDEM) and iii) GCM values. Over 2400 LiDAR project tiles were used, a subset of the current total LiDAR coverage that represents ∼1.5% of Canada. Fig. 8 illustrates the RMSE frequency distribution of DSMs, canopy heights, and DEMs to the benchmark LiDAR within the canopy corrected areas with >3 m estimated canopy height (GFC >0% and GCM >3 m and SLOPE ≤15%). By only looking at areas with forest coverage and where the canopy corrections were applied, we can better understand the performance of the canopy corrections applied to the DEMs.

Initially, all DSMs perform poorly, but the COP30m shows the best performance, with the narrowest RMSE frequency distribution (Fig. 8a). All canopy models exhibit similar performance, although applying a moving average window improved the C1W-NASADEM canopy model (Fig. 8b). After removing the canopy, the C1W-NASADEM performs comparably to the FABDEM (Fig. 8c), albeit with a slightly broader RMSE frequency distribution. The sharp, tall peaks in the RMSE frequency distribution indicate that both DEMs align well with the LIDAR through-canopy benchmark. Following canopy correction, and excluding FABDEM due to commercial licensing constraints, the C1W-NASADEM was found to be as the best-performing DEM product for forested areas in Canada (Fig. 8d), showing significant improvement over the original DSM and COP30m DSM.

### 4.13 Elevation performance against LiDAR benchmarks

To evaluate the performance of the DEMs, all datasets were compared to the benchmark LiDAR DEM (through-canopy, true bare-earth surface) using the same > 2400 project tiles previously mentioned, but coverage areas with and without canopy. By including areas with and without canopy, we can better understand the overall performance of the DEMs.

**Fig. 9:**
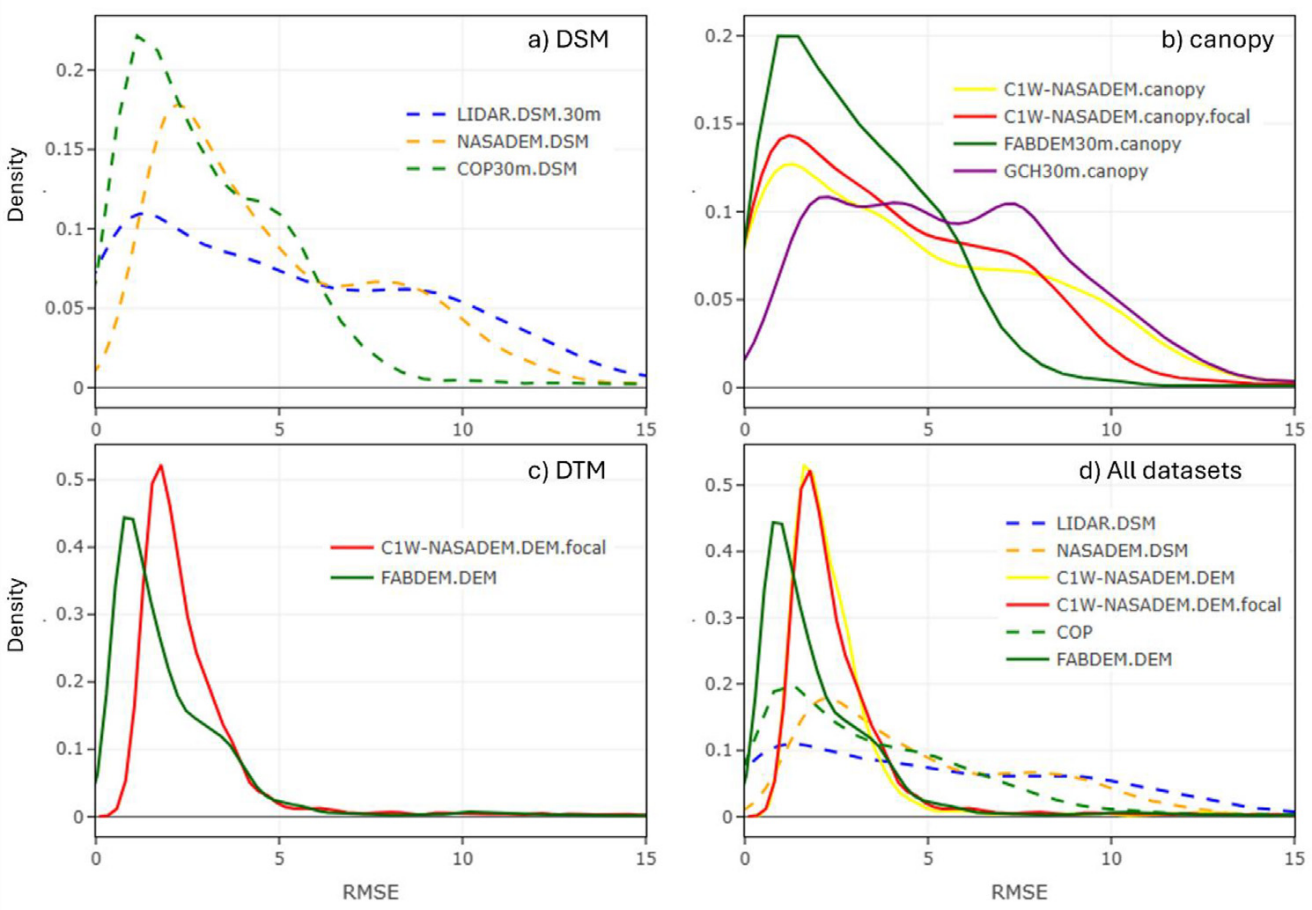
A comparison of datasets to the LiDAR benchmarks. DSMs (a) were compared to the LiDAR with top-of canopy included. Canopy models (b) are compared to the LiDAR canopy heights. DTMs (c) were compared to the LiDAR with ground surface (through-canopy). All datasets are compared to the LiDAR ground surface (through-canopy; d).

Four comparisons of model performance benchmarked to the LIDAR DEM coverage are presented (Fig. 9). The DSM comparison shows the relative biases due to canopy biases, where COP30m performs the best (Fig. 9a). When including the aggregate of areas with and without canopy, all canopy models have similar performance, with FABDEM being the best (Fig. 9b). After canopy-correction and ignoring the FABDEM (commercial licensing), the C1W-NASADEM is superior to COP30m DSM (Fig. 9c and Fig. 9d).

Overall, the C1W-NASADEM and associated canopy-correction model perform very well, greatly reducing the relative error between the original DSM and LiDAR benchmarks.

These corrections substantially enhance DEM accuracy, particularly in low-relief and moderately forested regions. However, performance declines in complex terrain with steep slopes, where canopy corrections are less effective. Continued refinement of canopy correction methods and targeted validation in underrepresented ecozones will further enhance DEM reliability for national-scale hydrological modelling.

### 4.14 Geographic terrain comparison

**Fig. 10:**
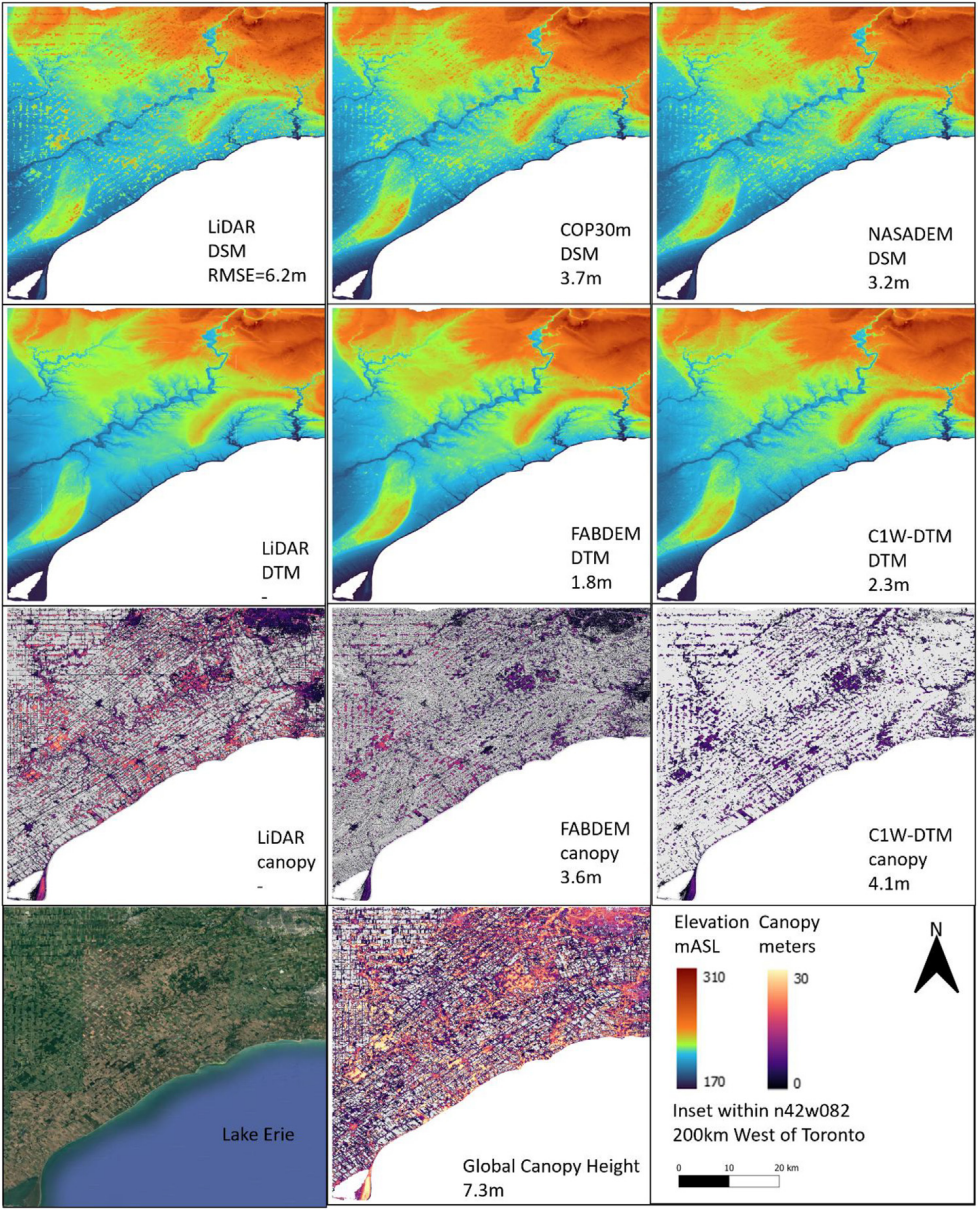
Area of southern Ontario, Chatham-Kent township, comparing the DSM (top row), DTM (2nd row) and canopy (3rd row) of the LiDAR (left column), COP30m/FABDEM (middle column) and NASADEM/C1W (right column). Included are the satellite imagery and Global Canopy Model (bottom row). The numbers below the inset titles are the RMSE between the respective dataset and the LiDAR DTM or canopy benchmark.

**Fig. 11:**
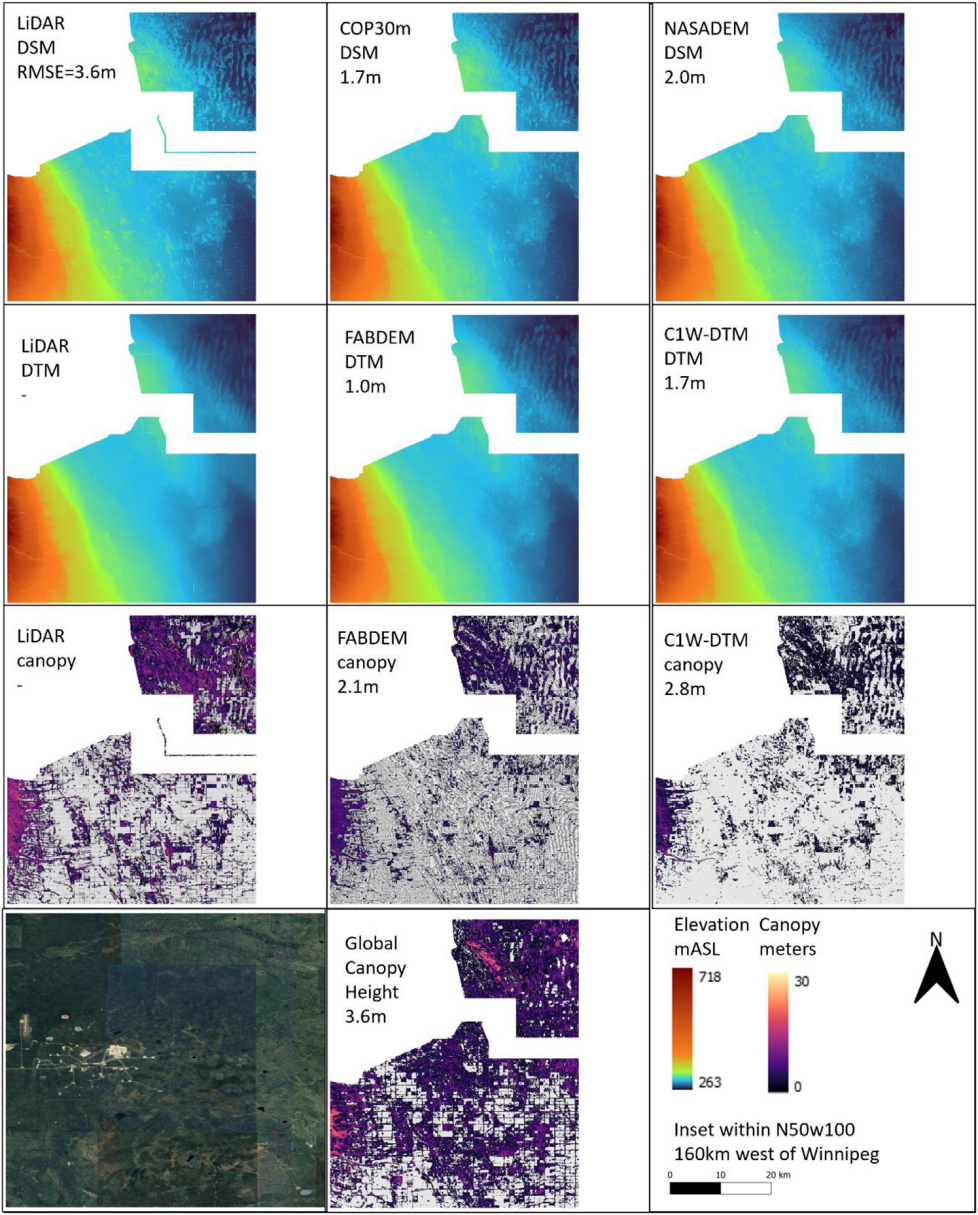
Area west of Winnipeg, Manitoba comparing the DSM (top row), DEM (2nd row) and canopy (3rd row) of the LiDAR (left column), COP30m/FABDEM (middle column) and NASADEM/C1W (right column). Included are the satellite imagery and Global Canopy Model (bottom row). The numbers below the inset titles are the RMSE between the respective dataset and the LiDAR DTM or canopy benchmark.

**Fig. 12:**
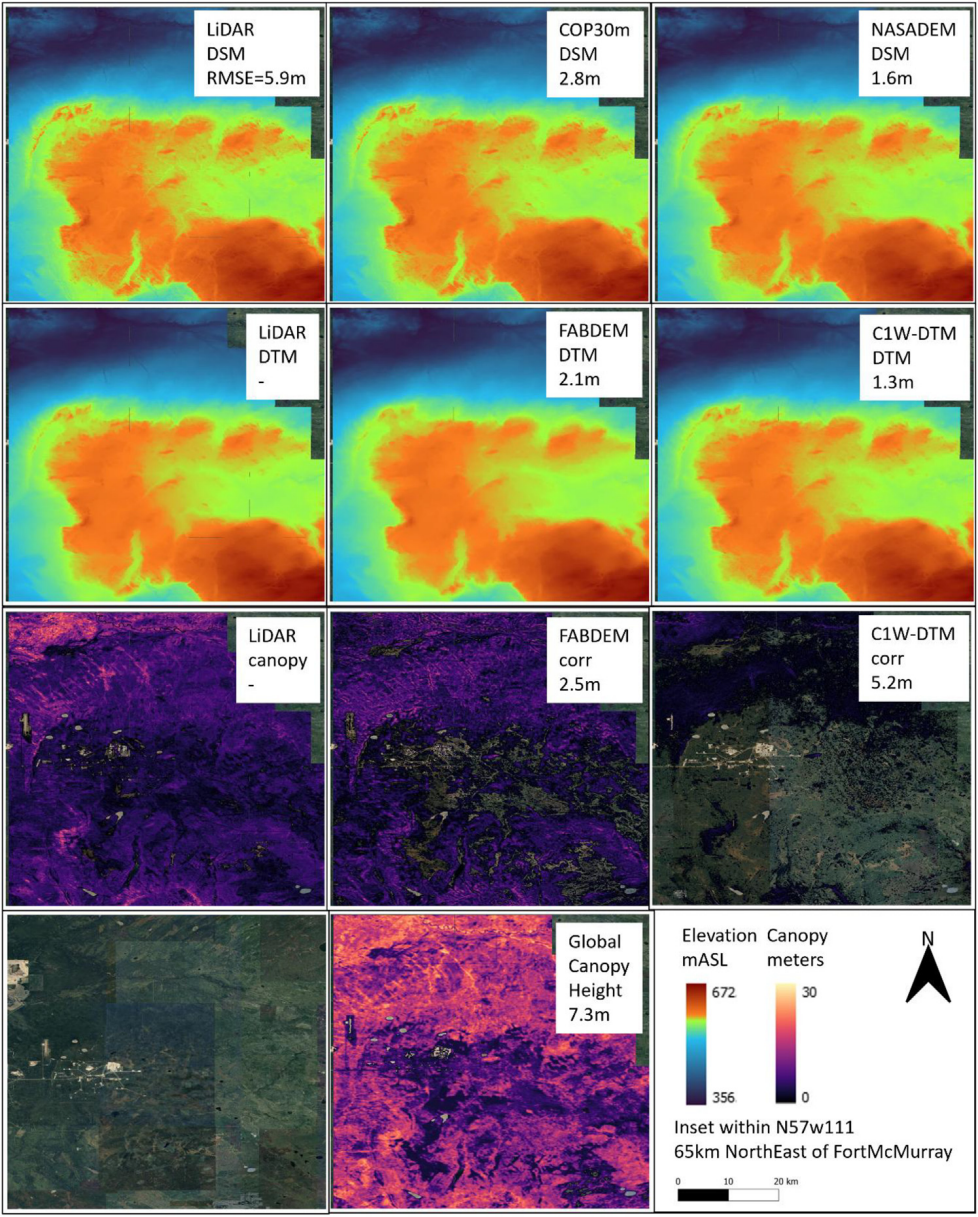
Area northeast of Fort McMurray, Alberta comparing the DSM (top row), DEM (2nd row) and canopy (3rd row) of the LiDAR (left column), COP30m/FABDEM (middle column) and NASADEM/C1W (right column). Included are the satellite imagery and Global Canopy Model (bottom row). The numbers below the inset titles are the RMSE between the respective dataset and the LiDAR DTM or canopy benchmark.

**Fig. 13:**
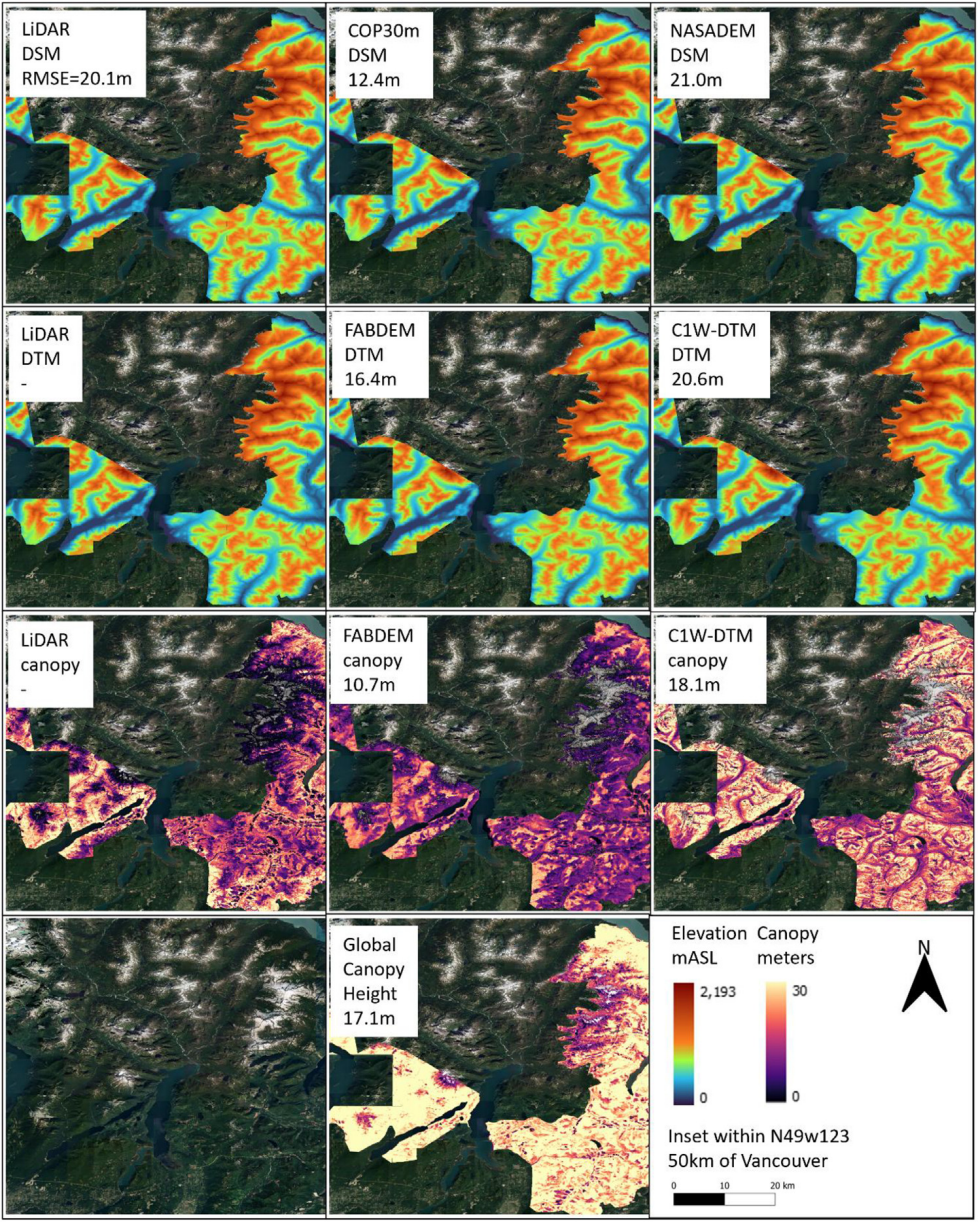
Area east of Vancouver, British Columbia, comparing the DSM (top row), DEM (2nd row) and canopy (3rd row) of the LiDAR (left column), COP30m/FABDEM (middle column) and NASADEM/C1W (right column). Included are the satellite imagery and Global Canopy Model (bottom row). The numbers below the inset titles are the RMSE between the respective dataset and the LiDAR DTM or canopy benchmark.

To further evaluate the C1W-DTM accuracy, four geographically separated and diverse areas (topography, land use, land cover), where DEM LiDAR benchmarks are available, were selected to compare the NASADEM DSM and NASADEM DTM (canopy corrected) pairing (Table 3). The COP30m and FABDEM dataset pairs are also included in the comparison to contrast the relative performance. The four areas were i) agricultural southern Ontario, ii) glaciofluvial delta landscape west of Winnipeg, iii) boreal forest northeast of Fort McMurray and iv) coast mountains east of Vancouver (Fig. 10, Fig. 11, Fig. 12, Fig. 13).

**Table 3:** Comparison of the geographic, land cover and drainage characteristics of the four test areas.

Location	Lat/ Long Tile	LiDAR Area (km^2^)	Physio-graphic domain	Relief	Drainage	Landcover	Mean DSM RMSE (m)	Mean DEM RMSE (m)	Mean DSM RMSE forested-areas only (m)	Mean DEM RMSE forested-areas only (m)
southern Ontario	n42 w082	4630	St Lawrence lowlands	Low	Low	Agricultural	3.2	2.3	6.2	2.7
west of Winnipeg	n50 w100	4133	interior plains	Low	Moderate	Agricultural	2.0	1.7	3.8	1.9
north east of Fort McMurray	n57 w111	1148	interior plains Boreal Forest	Moderate	Moderate	Boreal forest	1.6	1.3	3.4	2.6
east of Vancouver	n49 w123	1438	Western Cordillera	High	High	Barren rock	21.0	20.6	21.2	21.0

For an area in southern Ontario, north of Lake Erie in the Chatham-Kent township with low relief (120 m), extensive glacial lacustrine sediment, extensive agricultural fields with isolated wood lots and low-order drainage (Fig. 10). Canopy corrections performed well, where the mean RMSE between C1W-NASADEM and LiDAR DEM decreased from 3.2 m (DSM) to 2.3 m (DEM; Table 3). This relatively large decrease in RMSE is likely due to a combination of low relief and easily identifiable forests, which is favourable for canopy correction algorithms.

An area 160 km west of Winnipeg and northeast of Brandon, within the Assiniboine delta, is an abandoned glaciofluvial delta with low relief sand surface incised by moderate drainage and with extensive agricultural fields. Vegetation is sparse within the natural landscape outside the agricultural footprint. A modest improvement in RSME from 2.0 to 1.7 meters (15%) (Fig. 11) is likely reflective of the sparse vegetation but a well performing canopy correction, given the similar RMSE in canopy between NASADEM and FABDEM (2.8 and 2.1 m, respectively; Table 3).

The area 65 km north-east of Fort McMurray is in the vicinity of the Suncor Firebag village and part of the McMurray Lowlands of the interior plains. The landscape has moderate relief and land cover is boreal forest wetland dominated (Fig. 12). With the canopy correction, the RMSE had a modest improvement from 1.6 to 1.3 m, likely due to a combination of the high vegetation density, shorter canopy height and moderate relief.

A contrasting site 50 km east of Vancouver in the mountainous terrain of Alloutte and Stave lakes has relief of >2500 m, with mountainous slopes, forest cover at low elevations and barren rock above ∼1500m. Here, canopy corrections caused a slight decrease in RMSE from 21.0 to 20.6 m (Fig. 13) likely due to the high topographic slope (>15%) in which the up-sampled details in the MERIT corrections were lost or the canopy corrections were not considered.

Overall, the canopy corrections applied to the C1W-NASADEM are significant improvements to its performance. These corrections cover approximately 43% of the Canadian landscape below N60 latitude (defined as areas with canopy corrections >3m and GFC >0% and GCM >3 m and SLOPE ≤15%) with a mean canopy height of 5.5 meters, (Fig. 8b). The canopy corrections are corroborated by both the FABDEM AND GCM datasets, which have similar mean canopy heights in the LiDAR benchmark comparisons presented here (Figs. 8b, Fig. 10, Fig. 11, Fig. 12, Fig. 13). Notably, the canopy corrections were developed using a consistent and standardized methodology - an important objective in building the national-scale C1W-DTM. This contrasts with products like the MRDEM, which apply two different approaches for canopy correction.

The Canada1Water Digital Terrain Model with Lake Bathymetry (C1W-DTM) meets the need for a bi-national, harmonized dataset. By integrating the highest resolution DEMs available with digital lake bathymetry, this model delivers superior topography with enhanced spatial resolution, consistent coverage, and improved vertical accuracy across Canada. This improved DTM is a tool for various applications, including hydrological modelling and natural resource management, facilitating more accurate and informed decision-making.

## Limitations

The C1W-DTM was designed specifically to meet the needs of C1W and its primary objective: to establish a framework for national-scale hydrological modelling under a changing climate. The current model is comprehensive; however, additional enhancements and processing could further improve the C1W-DTM. For instance, addressing seamlines by smoothing or removing them would improve terrain continuity. Refining the canopy bias corrections would directly enhance the final DEM and DTM elevations, providing a more accurate representation of the canopy height. Furthermore, given Canada's relatively low urban footprint (∼2% of the landscape), no building bias correction has been applied to the C1W-DTM. Developing a building height model to correct for these biases would contribute to even further improvement.

## Ethics Statement

The authors confirm that they have read and follow the ethical requirements for publication in Data in Brief. This work does not involve human subjects, animal experiments, or any data collected from social media platforms.

## CRediT authorship contribution statement

**Eric D. Kessel:** Methodology, Data curation, Visualization, Writing – original draft. **Steve K. Frey:** Conceptualization, Writing – review & editing. **Hazen A.J. Russell:** Conceptualization, Writing – review & editing.

## Data Availability

(Zenodo).Canada1Water Digital Terrain Model with Bathymetry dataset (Original data)
